# Dermatomyositis presenting with symptomatic dermographism and raised troponin T: a case report

**DOI:** 10.4076/1752-1947-3-7319

**Published:** 2009-07-14

**Authors:** Kartini F Rahim, Robert S Dawe

**Affiliations:** 1 Department of Dermatology, Level 8, Ninewells Hospital, Dundee DD1 9SY, UK

## Abstract

**Introduction:**

Dermatomyositis is an important inflammation of skin and muscles. Generalised itch is frequent in the condition; however, symptomatic dermographism has not previously been reported as a presenting feature.

**Case presentation:**

A 32-year-old Caucasian Scottish woman was diagnosed with dermatomyositis after initial presentation with symptomatic dermographism. No underlying neoplasm was found and her condition was successfully treated with systemic corticosteroids and high-dose human immunoglobulin infusions. At presentation, her troponin T and creatine phosphokinase enzymes were highly raised.

**Conclusion:**

Symptomatic dermographism may be a presenting feature of dermatomyositis. Dermatomyositis is one of many conditions that can result in a raised troponin T.

## Introduction

When it presents in its classical form, dermatomyositis is easy to diagnose, with typical redness and scaling overlying the knuckles, purple-red discolouration especially about the upper eyelids and often associated with some eyelid oedema, redness over the upper back and shoulders (the "shawl sign") along with a predominantly proximal upper and lower limb muscle weakness. However, when it presents without this complete picture, diagnosis can be far harder. It is an important condition to diagnose, because it can in itself be a life changing and sometimes life threatening condition, and because when occurring with onset in adulthood it is important to look for a possible underlying neoplasm. Generalised itch is well recognised in the dermatological literature [1,2] as an important feature of dermatomyositis, although less attention is given to itch as a symptom in standard general medical textbooks [3]. However, dermographism (linear urticaria in response to stroking the skin) has not been previously described as a feature of this disease.

## Case presentation

**Figure 1:**
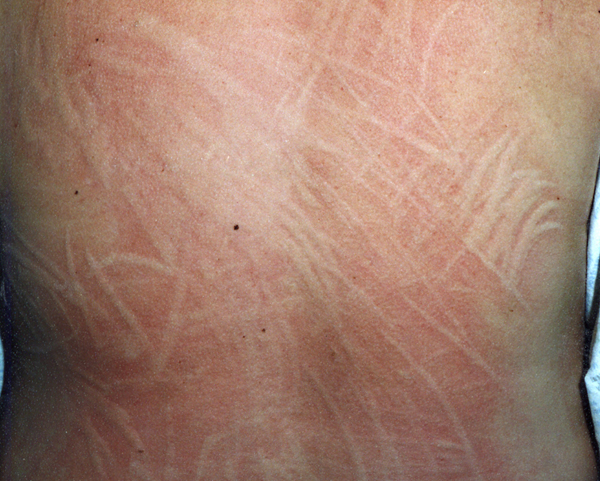
**Dermographic urticaria resulting from pressure through clothing**.

A 32-year-old Caucasian Scottish woman was attending thrice weekly for a second course of narrowband ultraviolet B (UVB) phototherapy for severe dermographism (Figure 1). Eight months earlier, a similar course of treatment resulted in complete clearance of her dermographism. It had, however, recurred rapidly over 2 months. Antihistamines were of minimal benefit, hence the re-referral for phototherapy. At the time of presentation to us, she had a slightly raised alanine aminotransferase (ALT) and alkaline phosphatase but investigation for possible explanations of these findings, and an underlying cause of generalised itch might be exacerbating her dermographism, was unrevealing. On her twelfth attendance at the phototherapy unit, she reported that she was being investigated for possible heart disease following an admission to an Acute Medical Unit after she developed chest pain after a fall on an icy pavement. A chest X-ray and electrocardiogram had not shown any abnormalities and an exercise tolerance test was awaited. We requested her blood test reports which revealed a high creatine phosphokinase (CK) of 6195U/L (reference range: 30-120) and troponin T (Elecsys Modular Analytics E170 [Roche Diagnostics]; reagent kit Cat. No 12017644) of 0.14μ g/L (reference range 0-0.01).

**Figure 2:**
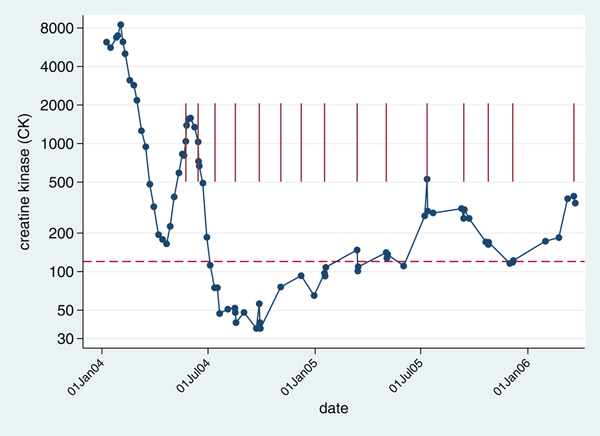
**Change in creatine kinase (U/L) over time**. The vertical maroon lines indicate IVIG infusions. The horizontal dashed line indicates the upper limit of the reference interval for CK.

She was reassessed. Her dermographism was improving but she had developed confluent erythema and slight hyperkeratosis between and over the dorsae of her fingers. She had dilated nailfold capillaries on the little finger of her right hand. Specific questioning elicited a history of proximal arm discomfort when lifting her young child. She had slight difficulty when asked to rise from a chair without using her arms. The combination of these skin changes, the muscle symptoms and her elevated muscle enzymes suggested a diagnosis of dermatomyositis. We admitted her for rest and to expedite investigations. Histopathology of a muscle biopsy and electromyography features supported the impression of inflammatory myositis consistent with dermatomyositis. A transthoracic echocardiogram did not show any abnormalities. No malignancy was found in her history, or during examination and investigation. Oral systemic corticosteroids were started.

At outpatient review, soon after discharge, her dermatomyositis was responding well to treatment. We requested troponin T as well as CK. Her CK had fallen to 3000 and her troponin T to 0.10. These results were telephoned through to her general practice (before being returned to us in hospital) and, alarmed by the high troponin T, a locum general practitioner referred her urgently to the Acute Medical Unit (AMU) that night. One of us, who had reviewed her earlier that day in outpatients, overheard a telephone enquiry about her seeking her case notes from the AMU to our ward. On assessment, there was nothing new of concern and, after reassurance that her blood test results were improving, she was allowed home.

After an initial response to oral corticosteroids alone, the rate of improvement in her condition slowed. Therefore, high-dose human intravenous immunoglobulin (IVIG) therapy was added [4]. Shortly after the first IVIG infusion, her symptoms and biochemical features started to improve quickly (Figure 2). A few months after starting regular IVIG, her CK reached near normal population values and her skin and muscle symptoms and signs resolved. Her dermographism completely resolved, and has not recurred. Her condition continued to gradually improve. The IVIG infusion frequency was reduced and then infusions stopped. She was maintained on a low dose of prednisolone during a pregnancy, 4 years after her diagnosis. This had to be increased to 10 mg daily for a few months after delivery, because of increased activity of her disease, but has now been reduced again.

## Discussion

Possibly, our patient's symptomatic dermographism was coincidental to her presentation with dermatomyositis. Dermographism is common, affecting about 5% of people [5]. Her dermographism was particularly severe, requiring UVB phototherapy [6]. We are suspicious that her symptomatic dermographism was related to her dermatomyositis, possibly through the mechanism of generalised itch leading to rubbing and scratching and so making much more evident what could otherwise have been only mild dermographism. Although not reported in the published literature, we have an impression that dermographism is more frequent, perhaps affecting almost half of patients, in dermatomyositis than would be expected by chance. Early during the course of her illness, she was worried, we think unnecessarily, by an emergency admission which was prompted solely by a blood test result report (the troponin T). Elevated troponin T has been reported as a feature of dermatomyositis, possibly due to cardiac muscle involvement [7]. Also, more recent published correspondence has re-emphasised that troponin T is not a specific test for ischaemic cardiac damage [8]. Our management of this patient's dermatomyositis was fairly standard, although influenced by the fact that she wanted to have more children, so we relied on prednisolone and, initially, IVIG infusions rather than other systemic therapies. If we had needed to use other therapy, then azathioprine would have been an option, as a drug that does not seem to be teratogenic [9].

## Conclusion

This case report illustrates two things. First, we report symptomatic dermographism as the presenting feature of dermatomyositis. Second, this case should be a reminder to doctors from all medical specialities that while the relatively recent widespread availability of troponin T assays has been an important advance, troponin T can be raised for reasons other than acute cardiac ischaemia.

## Abbreviations

ALT: alanine aminotransferase; AMU: acute medical unit; CK: creatine phosphokinase; IVIG: high-dose human intravenous immunoglobin; UVB: ultraviolet B.

## Consent

Written informed consent was obtained from the patient for publication of this case report and any accompanying images. A copy of the written consent is available for review by the Editor-in-Chief of this journal.

## Competing interests

The authors declare that they have no competing interests.

## Authors' contributions

RD initially saw, and followed up this patient throughout the course of her illness. KR saw the patient during some review visits and wrote the initial drafts of the manuscript. Both authors read and approved the final manuscript.
